# Supplementary figures and images for: Expression-based species deconvolution and realignment removes misalignment error in multispecies single-cell data
Source: BMC Bioinformatics. 2022 May 2;23:157. doi: 10.1186/s12859-022-04676-0 (PMC9063264; doi:10.1186/s12859-022-04676-0)

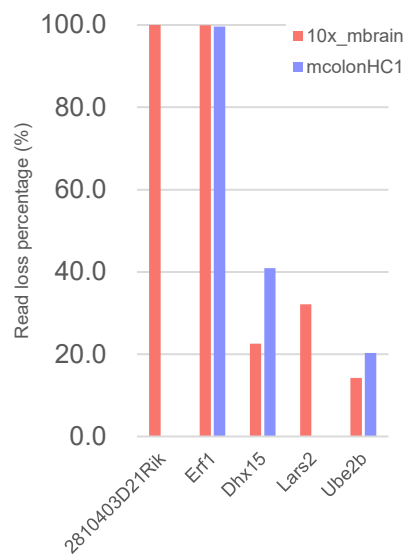

**Additional file 2. Read loss percentage of the most misaligned genes in mouse**

Supplement: Supplementary file 2 — Additional file 2. Read loss percentage of the most misaligned genes in mouse. [file 12859_2022_4676_MOESM2_ESM.pdf]

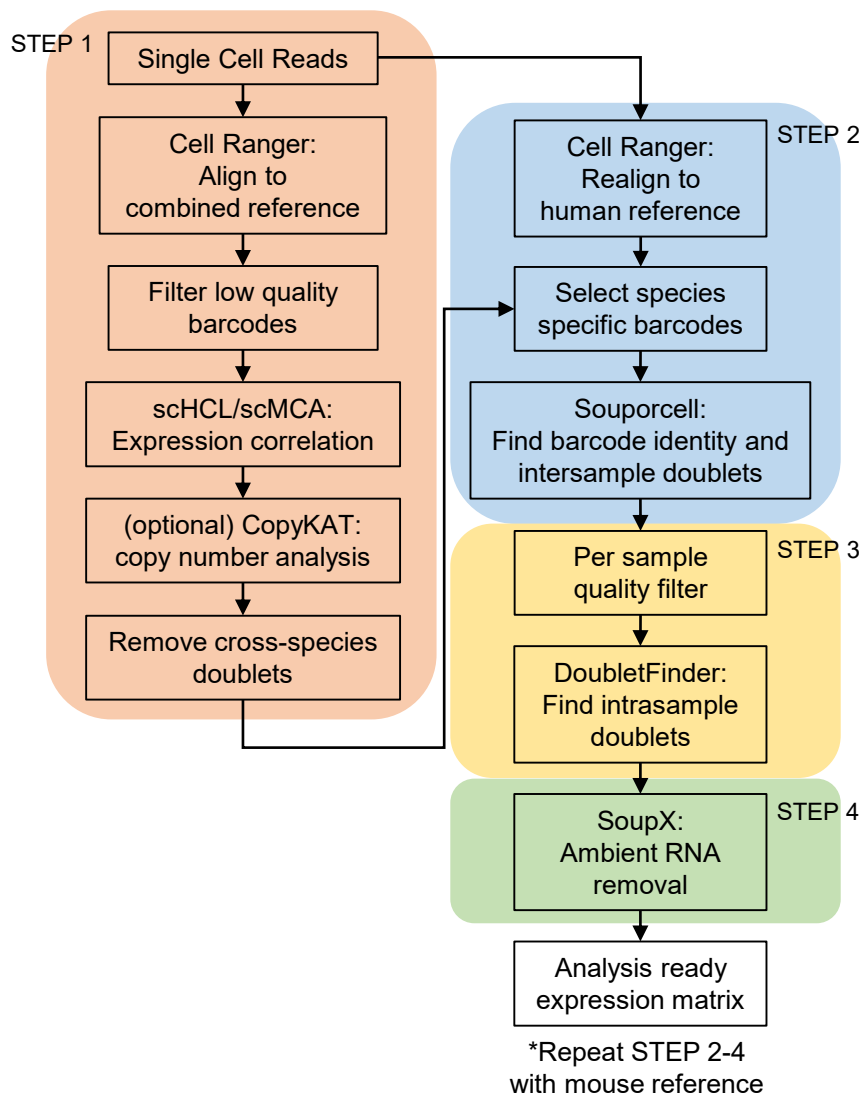

**Additional file 4. Flowchart of REMS pipeline**

Supplement: Supplementary file 4 — Additional file 4. Flowchart of REMS pipeline. [file 12859_2022_4676_MOESM4_ESM.pdf]

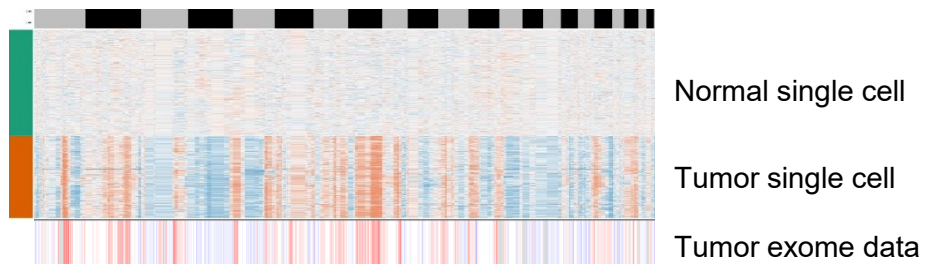

**Additional file 7. Copy number from single-cell and exome data**

Supplement: Supplementary file 7 — Additional file 7. Copy numbers from single cell and exome data. [file 12859_2022_4676_MOESM7_ESM.pdf]
