# Supplementary material for: Expression-based species deconvolution and realignment removes misalignment error in multispecies single-cell data
Source: BMC Bioinformatics. 2022 May 2;23:157. doi: 10.1186/s12859-022-04676-0 (PMC9063264; doi:10.1186/s12859-022-04676-0)
Supplement: Supplementary file 5 — Additional file 5. MAP of human lung cells processed using Cell Ranger annotated by (A) library and (B) cell type. UMAP of human lung cells processed using REMS pipeline annotated by (C) library and (D) cell type. [file 12859_2022_4676_MOESM5_ESM.pdf]

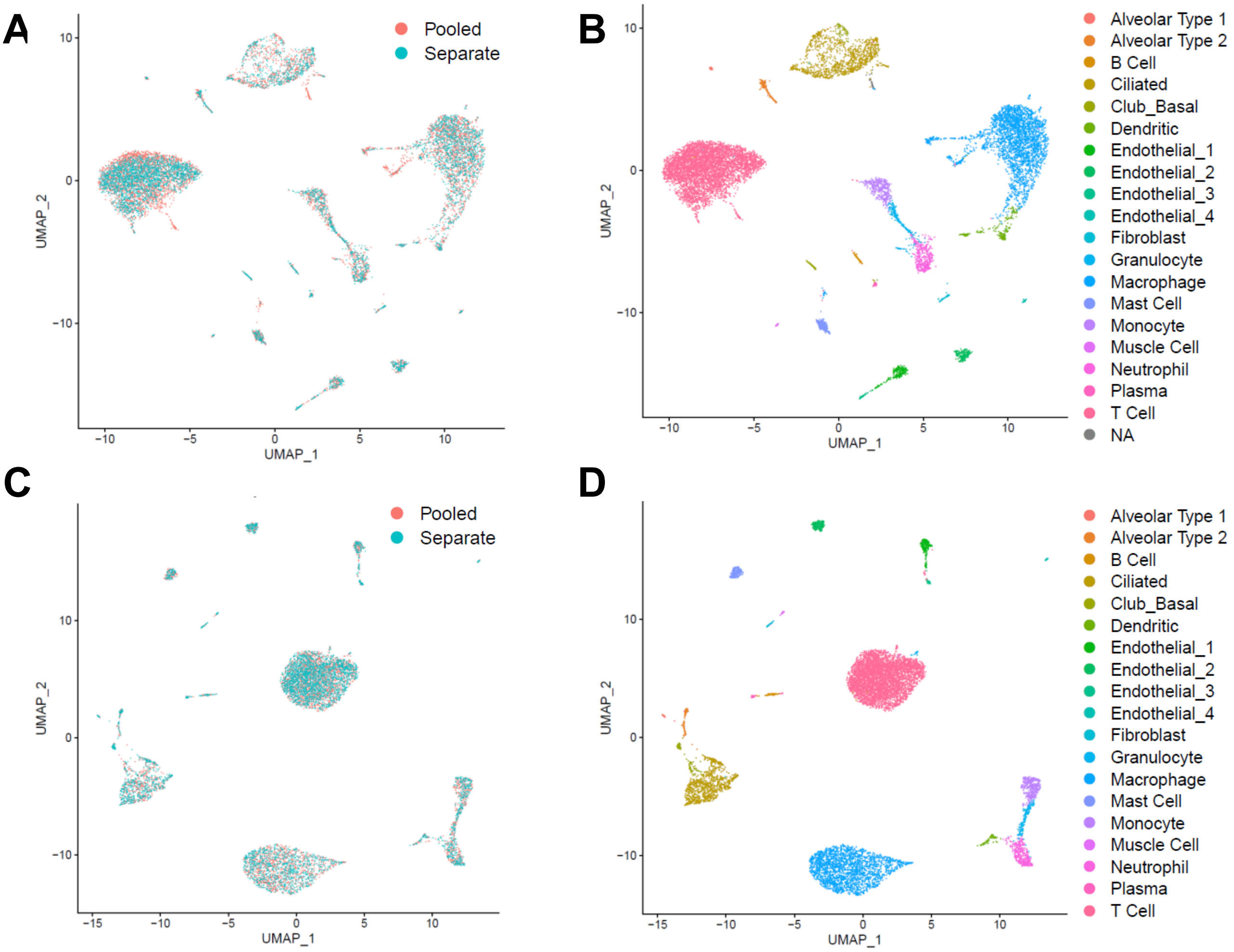

Additional file 5. UMAP of human lung cells processed using Cell Ranger annotated by (A) library and (B) cell type. UMAP of human lung cells processed using REMS pipeline annotated by (C) library and (D) cell type.
