# Supplementary material for: Expression-based species deconvolution and realignment removes misalignment error in multispecies single-cell data
Source: BMC Bioinformatics. 2022 May 2;23:157. doi: 10.1186/s12859-022-04676-0 (PMC9063264; doi:10.1186/s12859-022-04676-0)
Supplement: Supplementary file 6 — Additional file 6. Comparison of “REMS with copy number filter” and “REMS without copy number filter”. [file 12859_2022_4676_MOESM6_ESM.pdf]

A

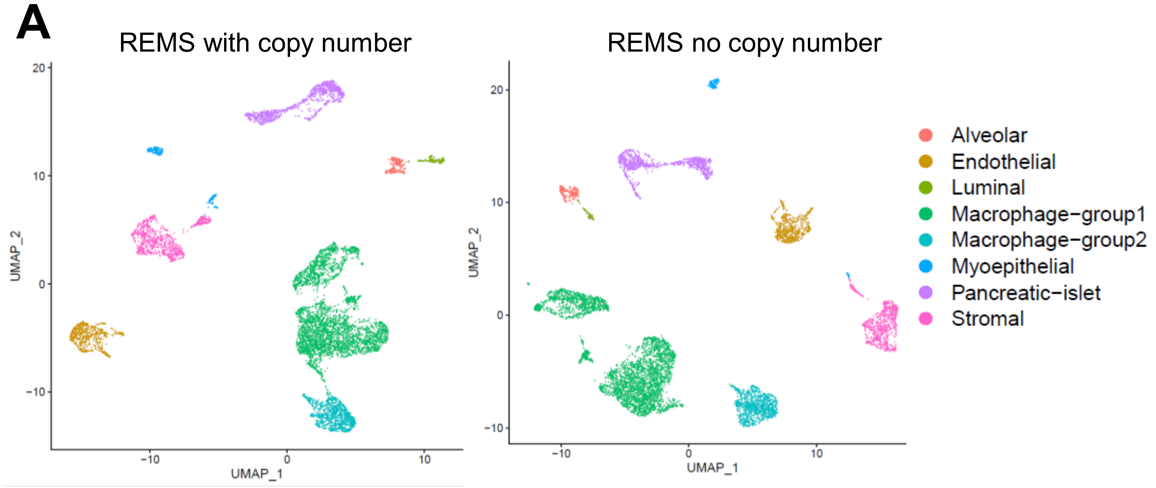

B

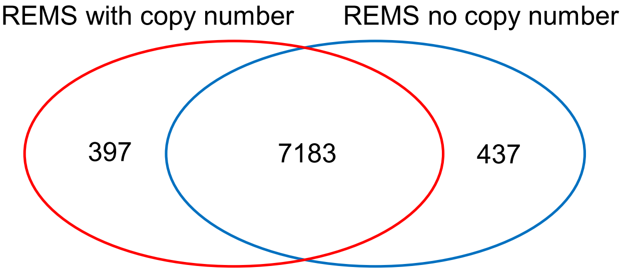

count	Filter reason (other pipeline)
47	Quality fail: low feature count
71	Quality fail: low read count
1	Souporcell identification fail
3	Souporcell doublets
275	Doubletfinder doublets

count	Filter reason (other pipeline)
210	Copy number filter
4	Quality fail: low feature count
1	Quality fail: low read count
6	Souporcell doublets
216	Doubletfilnder doublets

C

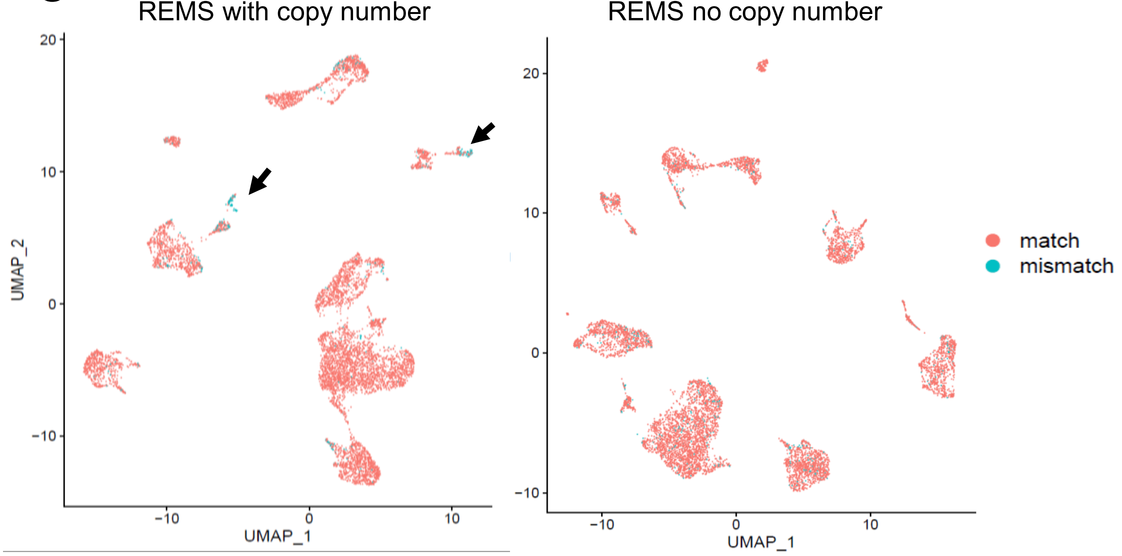

Additional file 6. Comparison of “REMS with copy number filter” and “REMS without copy number filter”

(A) Celltype annotation of UMAP for each pipeline, all celltype clusters were found for both pipelines. (B) Venn diagram with filter reason from the other pipeline for the barcodes which are not in common. “REMS with copy number filter” had 397 among 7580 barcodes which were not present in the other pipeline, and “REMS without copy number filter” had 437 among 7620 barcodes which were not present the other pipeline. (C) UMAP of cells which are not included in the other pipeline, two clusters with high mismatch are marked with black arrow.
Cells present in “REMS with no copy number filter” which were filtered in the other pipeline due to copy number filter were spread throughout all cell clusters. We suggest that this discordance is due to highly conservative filter criteria. Other than copy number filter, DoubletFinder caused the most disparities. In “REMS without copy number filter”, doublets called by DoubletFinder were concentrated in two clusters; myoepithelial cluster and luminal cluster. These celltypes were found in an independent patient derived xenograft single cell (data not shown) which we have not included in the manuscript due to poor human cell sequence quality. Doublets called by DoubletFinder also had high scMCA correlation values (average correlation value for myoepithelial singlets: 0.529, myoepithelial doublets: 0.523; luminal singlets: 0.565, luminal doublets: 0.543). For the above reasons, we believe these cells are false positive intra-sample doublets, and with manual inspection, may be salvaged from DoubletFinder filters.
